# Experimental Investigation on High Capacity Stove-Powered Thermoelectric Generator Incorporated with a Novel Heat Collector

**DOI:** 10.3390/ma15072382

**Published:** 2022-03-23

**Authors:** Xiaoxiao Ru, Guoneng Li, Youqu Zheng, Wenwen Guo, Yuanjun Tang

**Affiliations:** 1 Chinese-German School, Zhejiang University of Science and Technology, Hangzhou 310023, China; ruxiaoxiao1981@126.com; 2 Department of Energy and Environment System Engineering, Zhejiang University of Science and Technology, Hangzhou 310023, China; zyq888@zust.edu.cn (Y.Z.); 115030@zust.edu.cn (W.G.); tang@zust.edu.cn (Y.T.)

**Keywords:** thermoelectric generator, biomass stove, thermoelectric efficiency, theoretical analysis

## Abstract

It is vital to supply necessary electric power during natural disasters and in deprived regions. A novel heat collector is proposed to improve the capacity of the stove-powered thermoelectric generator (SPTEG). Enclosed combustion walls are constructed with four W-shape copper plates, and act as a whole to be an exceptional heat collector, which was not previously reported in TEG studies. Forty TE modules are installed and two DC–DC converters are employed to stabilize the electric power. Owing to the novel heat collector, the generated electric power reaches 0.024 W/K per unit temperature difference for an individual TE module, which is 200% higher than the previous record (0.008 W/K) when forty TE modules are incorporated. The proposed SPTEG is able to generate a net electric power of 119 W, which is considerably larger than the previous record (75.2 W). The corresponding TE efficiency reaches 3.12%, which is measured at a temperature difference of 140 °C. The startup performance, power load feature, and cooling water flow rate of the SPTEG are studied in detail. Furthermore, one-dimensional theoretical analyses are conducted to explore the SPTEG performance. The theoretical electric power agrees well with the experimental data when DC–DC converters are not involved. Applying DC–DC converters to stabilize the electric power will alter the impendence of the SPTEG, resulting in much lower electric power output than that without DC–DC converters.

## 1. Introduction

Fossil fuels are the dominant source of energy promoting human civilization. Meanwhile, burning fossil fuels leads to serious greenhouse effects [1]. Therefore, alternative resources are proposed, such as solar, wind, and biomass. Biomass stoves are used widely to meet energy needs by billions of people because no advanced technology is involved; only a simple stove [2]. Burning biomass has zero CO_2_ emissions in its life cycle, but CO and particle pollutants from stoves are known to be harmful [3]. Possible solutions to reduce these pollutants include using a blower to provide sufficient combustion air [4]. Therefore, it would be beneficial if a certain amount of electricity can be generated by the stove itself. Thus, powering up a blower becomes possible for stoves in off-grid areas. Meanwhile, 1.1 billion people, about 15% of the worldwide population, still live in off-grid areas [2], mostly located in deprived regions. Besides, powering up small medical devices, cell phones, and small lamps is essentially important when natural disasters occur.

A stove-powered thermoelectric generator (SPTEG) can be one solution to provide such an amount of electricity. The idea of an SPTEG is incorporating a sufficient number of thermoelectric (TE) modules into the biomass stove while cooking and heating still work simultaneously [5]. An SPTEG utilizes the See beck effect to generate electricity, and the See beck effect illustrates the orderly flow of electrons driven by the temperature difference between the hot- and cold-ends of the TE materials. The major advantage of an SPTEG is the fuel sustainability locally, that is, any combustible matter can be used. Other advantages include little maintenance, weather independence, and little moving parts. However, drawbacks are obvious, which mainly refer to the low efficiency and high cost. The efficiency could be increased by using advanced TE materials. As reported recently, The *ZT* value ofBi_2_Te_3_/Sb_2_Te_3_ is 2.4 [6] and this value is 2.2 for PbTe [7]. Recently, Hinterleitner et al. [8] reported that a metastable thin-film Heusler alloy has a ZT value of over 5.0. Detailed information on various TE materials was presented in a recent review paper [9], and unfortunately, the abovementioned advanced TE materials are not ready for mass production. On the other hand, the high price will be reduced by extensive production. Therefore, the SPTEG has good prospects, attracting more and more attention in recent years from principle parameters [10], key issues [11], to extensive applications [12] and particular applications indeprived regions [13].

The TE generator (TEG) has been investigated in many previous studies, such as heat and power cogeneration [14], heat recovery from biomass gasifier [15], internal engine [16], and oil heater [17]. This work only presents a critical review of selected SPTEG experimental works. Experimental works are categorized as air-cooled SPTEG and water-cooled SPTEG. Detailed performances of these SPTEGs were summarized in Table 1, and a brief review is provided in the following paragraphs.

For air-cooled SPTEGs, the generated electric power in the SPTEG developed by Nuwayhid et al., was improved from 0.9 W [18] to 3.0 W [19], and then to 4.2 W [20]. No DC–DC converter (DDC) was employed, and the mass weight was approximately 40 kg [20]. Lertsatitthanakorn [21] developed a 2.4 W SPTEG. The corresponding TE efficiency is 3.2% at a temperature difference of 150 °C. Raman et al. [22] designed an SPTEG to reduce indoor air pollution and improve combustion efficiency. The maximum electric power was 4.5 W at a temperature difference of 240 °C. The SPTEG proposed by Najjar and Kseibi generated a maximum electric power of 7.8 W [23,24], and the influence of different fuel types on temperature distribution and power output was explored [24]. O’Shaughnessy et al. [25] ran a field test of SPTEG for 80 days in rural Malawi and concluded that the minimum daily electricity requirement is 3 Wh. Recently, commercial BioLite SPTEGs were shown to provide 2–5 W of electricity [26]. The mass weight of the BaseCamp is 8.16 kg, and is able to provide an electric power of 5 W. One of our works [27] demonstrated a new prototype of air-cooled SPTEG, which was able to generate a maximum electric power of 12.9 W, and the mass weight was 8.4 kg.

For water-cooled SPTEGs, Rinalde et al. [28] obtained 12.3 W based on their SPTEG. Champier et al. [29,30] proposed a new type of SPTEG, and 7.6 W was recorded at a TE efficiency of 2%. Montecucco et al. [31,32] successfully increased the electric power to 27 W in their water-cooled SPTEG, and the TE efficiency was 4–5%. Recently, Sornek et al. [33] developed a wood-fired SPTEG, which incorporates as many as forty TE modules. The generated electric power was 75.2 W with three TEG units (two water-cooled units and one air-cooled unit, 40 TE modules in total). Compared with air-cooled SPTEGs, water-cooled SPTEGs perform better than air-cooled ones because the heat capacity of water is much larger than that of air, which ensures excellent cold-end temperature uniformity. Hence, the water-cooling method is selected in the present work.

Surveying the above literatures, various types of SPTEG have been developed, indicating that SPTEG is a potential solution to provide electricity in off-grid areas and under emergency conditions. However, the generated electric power is less than 10 W for most previous SPTEGs, which restricts many possible demands, such as pumping water or powering up a laptop computer. Therefore, SPTEG should be improved to provide more electricity, e.g., 200 Wh per day [5].

In this work, a novel heat collector is proposed to improve the capacity of SPTEG, which facilitates the installation of as many as forty TE modules. The present study offers a novel prototype of water-cooled SPTEG, which provides a new strategy to augment power generation. In addition, the influence of DDCs on SPTEG performance is demonstrated. First, the SPTEG configuration and experimental setup are presented. Second, detailed performances including startup, power output, conversion efficiency, and cooling settings are shown and discussed. Third, a one-dimensional theoretical model was used to analyze the performance of the SPTEG. Finally, several conclusions were drawn.

## 2. SPTEG Configuration and Experimental Setup

### 2.1. SPTEG Configuration

**Figure 1:**
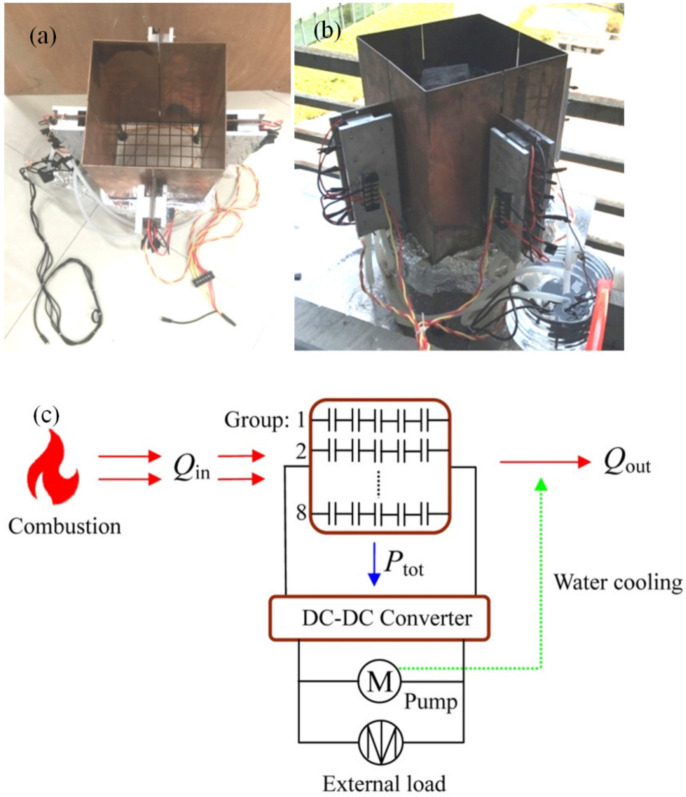
SPTEG configuration and electric circuit. (**a**) Top view of the SPTEG. (**b**) Side view of the SPTEG. (**c**) Schematic of the electric circuit for the present SPTEG with forty TE modules.

Figure 1 presents the SPTEG configuration and its corresponding electric circuit. Four copper heat-collecting plates (ChuangRui Co., Ltd., Hangzhou, China), forty TE modules (Sagreon Co., Ltd., Wuhan, China), eight water-cooled heat sinks (ChuangRui Co., Ltd., Hangzhou, China), four water pumps (Shenzhen Hengyuansheng Electronics Co., Ltd., Shenzhen, China), two DDCs (Shaibang Co., Ltd., Shenzhen, China), a porous fuel holder (ChuangRui Co., Ltd., Hangzhou, China), and a water container (ChuangRui Co., Ltd., Hangzhou, China) are assembled into an SPTEG. The enclosed combustion walls were formed by installing the abovementioned W-shaped heat-collecting plates, and four flat “ears” are also formed in the meantime to install TE modules. A 30 mm insulation layer was installed to decrease the heat loss from the combustion walls. Dimensions of the combustion chamber are 200 mm × 200 mm × 350 mm (height), and a fuel holder was installed to support the biomass combustion. Four iron feet with 50 mm height were fitted to support the whole SPTEG. Forty TE modules, type “SAGREON 12708”, were assembled on the abovementioned four “ears” equally spaced, and the dimensions of the TE module are 40 mm × 40 mm × 4.0 mm. The TE material is Bi_2_Te_3_, and its properties from the manufacturer are shown in Table 2. Every five TE modules were installed on one side of an “ear” as a group, and another five TE modules were fitted on the other side of the “ear” as another group. Therefore, there are eight groups, wiring the TE modules in series inside a group, and eight groups were connected to the DDCs in parallel. For each group of TE modules, a water-cooled heat sink, with dimensions of 40 mm × 12 mm × 200 mm, was employed and fastened by ten bolts.

The output voltage was conditioned to the required values for the water pumps and external load with two XL4016E step-down DDCs. The working voltage of the water pump can be adjusted between 4 V and 9 V, corresponding to a lift between 1 m to 2 m. The height between the top end of the water-cooled heat sinks and the water tank surface is 500 mm. Running water was supplied to a 2 L water tank, and the water after cooling was abandoned. In case the water is limited, the present SPTEG can be easily modified to a closed-loop water-cooled SPTEG with a certain number of blowers and a radiator. The weight of the whole SPTEG, including a fiberglass insulation layer and a water tank, is 17.6 kg.

The schematic of the SPTEG electric circuit is shown in Figure 1c. The heat-conducting plates absorb a part of the thermal energy released by flame (*Q*_in_), which is partially transformed into electric power (*P*_tot_) and the rest of the thermal energy is dissipated by the cooling water (*Q*_out_). The conditioned electric power supports water pumps, and the rest of the electric power can be utilized by external loads.

### 2.2. Heat Collector

The proposed heat collector consists of four identical W-shaped copper plates. There are several features: first, the heat collector provides eight identical flat plates to install forty TE modules (five TE modules on each side of a flat plate as a group), thereby ensuring large electric power generation. Second, this design ensures comparable temperature distribution in each group, which obviously reduces the number of DDCs because the generated voltage from each group of TE modules is comparable with each other, otherwise considerable internal power loss is unavoidable. Therefore, ten DDCs should be used instead of two in the present SPTEG. The temperature distribution is presented in Section 3.1. Third, a large heat absorption area was designed to collect sufficient heat flux inside the combustion chamber, which is critical when thermal radiation dominates the total heat flux [34]. Fourth, the present combustion chamber maintains a clean and tidy design without any protruding objects, which is beneficial during refueling. Fifth, this design provides a new clamping method, that is, the major contacting areas during clamping are located at the cold ends, which minimize the heat losses from hot ends through bolts.

Protruding objects (aluminum fins and copper bars) were employed in previous SPTEGs. As a consequence, limited heat flux can be collected due to the restricted area. Thus, only limited TE modules could be applied. Furthermore, these protruding objects lead to inconvenience during refueling, and the assembly between the abovementioned protruding objects and the heat spreader might cause abnormal functionality of SPTEGs [25].

### 2.3. Experimental Setup

**Figure 2:**
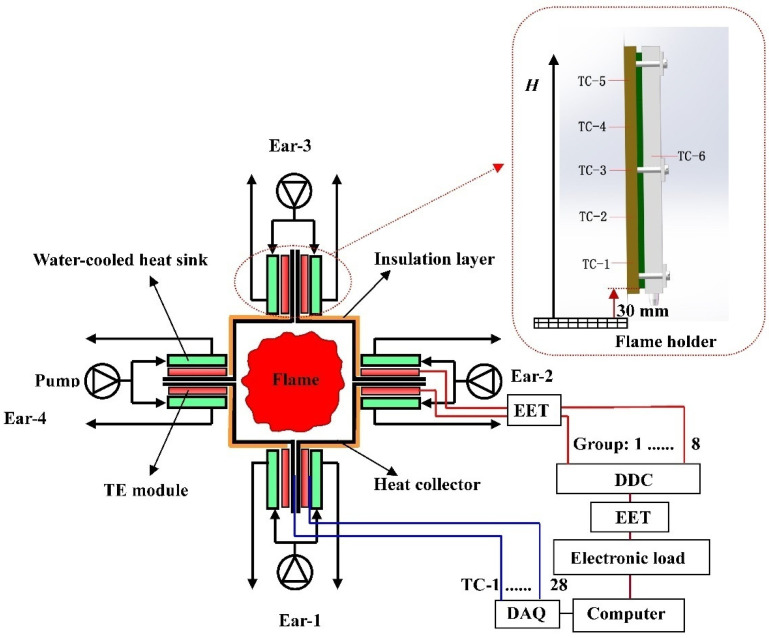
Experimental setup of the SPTEG incorporated with forty TE modules.

Seven thermocouples (Shangyi Co., Ltd., Shuanghai, China) with a diameter of 1 mm were installed on each “ear”, which is shown in the upper-right part of Figure 2. TC-7 is not shown, and it was located at the heat sink of the opposite TE group in a position similar toTC-6. Another two thermocouples were installed to measure the inlet and outlet cooling water temperatures. Thereby, a total of 30 thermocouples were installed in the whole SPTEG, and an Agilent 34970A (Agilent Co., Ltd., Santa Clara, CA, USA) was used to collect these temperature data. An electronic load instrument (Prodigit Co., Ltd., Taiwan, China), type Prodigit 3311F, was employed to measure the power load feature under the constant resistance mode. Two electric power testers (EETs, Juneng Xindi Electronic Technology Co., Ltd., Shenzhen, China), type JUWEI J7-7, were installed to measure the total electric power (*P*_tot_) and the line loss since a considerable magnitude of current was involved. The measuring ranges of the abovementioned thermocouple, electronic load, and EET are −200–400 °C, 0–60 V (300 W), and 1 V ≤ *U* ≤ 100 V with 0 A ≤ *I* ≤ 15 A, respectively. Besides, measuring errors are presented in Table 3.

### 2.4. Efficiency of the DDC

**Figure 3:**
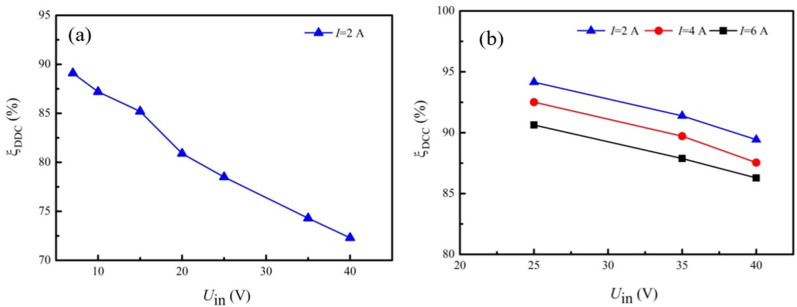
Efficiency of the DDC under various input voltages. (**a**) *U*_out_ = 6 V. (**b**) *U*_out_ = 19 V.

The DDC transform efficiency, *ξ*_DDC_, varied considerably because the input voltage and the output current were changing in different experimental cases. Figure 3 shows the DDC transform efficiency under various running parameters, which was measured in a separate experiment. In the present work, one DDC (DCC-1) powers up the water pumps at a voltage of 6.0 V. The other DDC (DCC-2) supplies electricity to external loads, and the output voltage was 19.0 V. As shown in Figure 3, the ratio of input voltage to output voltage is the major parameter affecting the transform efficiency of the DDC, while the output current is another important parameter. As shown in Figure 3, the transform efficiencies for DDC-1 and DDC-2 are above 70% and 85%, respectively. Concerning the maximum power point tracking (MPPT) DDC, no MPPT DDC performs apparently better than the present DDC, which was found after testing several types of MPPT DDCs. Therefore, regular DDCs were selected in the present work.

### 2.5. Fuel and Experimental Procedure

Charcoal of pine was employed for the present SPTEG tests. The lower heat value is 27.2 MJ/kg, and the ash fraction is lower than 4.6%. The inlet cooling water temperature is 29 °C, corresponding to hot summer days. The experimental procedures for a run of SPTEG include several steps, those are, step 1: turn on measuring instruments and initial all necessary recoding programs. Then, initial the combustion of charcoal (1.5 kg) with sufficient dry twigs. Step 2: perform all necessary tests (temperatures and electric powers) when the SPTEG reaches a steady state. Step 3: refuel the SPTEG until all required parameters are measured. Step 5: stop the SPTEG by dousing the fire.

## 3. Results and Discussion

### 3.1. SPTEG Startup and Temperature Distribution of Heat Collector

**Figure 4:**
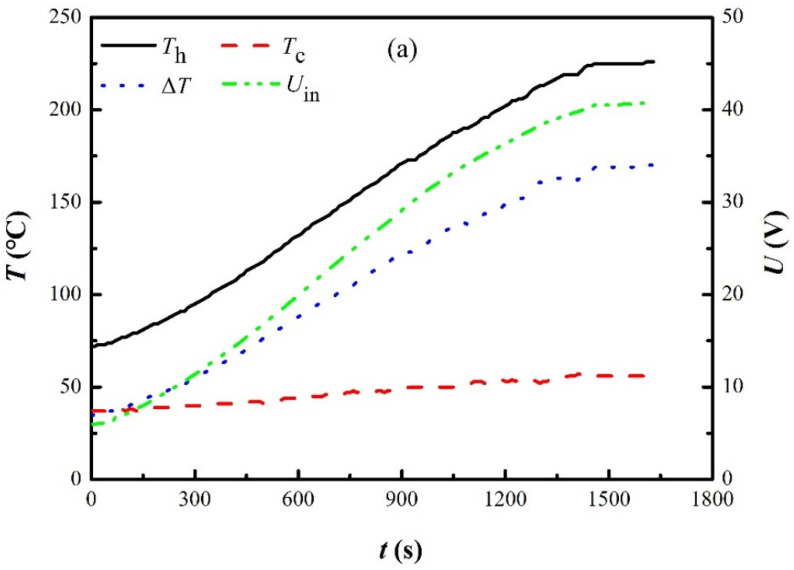

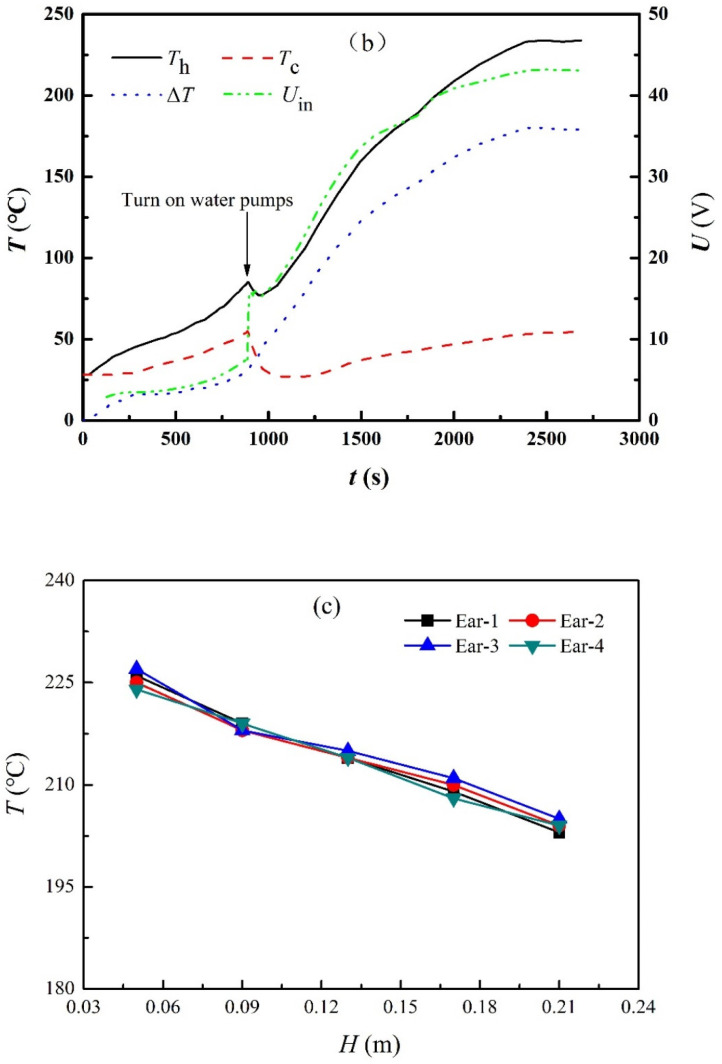
Parameter time history of SPTEG and temperature distribution of heat collector. (**a**) After switching the power source to the SPTEG. (**b**) Self-startup process. (**c**) Temperature variations in the height direction of the heat collector.

A battery was employed to power up water pumps until the open-circuit voltage generated by the SPTEG reached 8.0 V. The power source was switched to the SPTEG thereafter. The time needed (about 800 s) to switch the power source is different from case to case, depending on the growth of the combustion after ignition. Figure 4a shows the time history of the input voltage, temperature difference, and hot- and cold-end temperatures after switching the power source to the SPTEG. As shown in Figure 4b, a temperature difference of 32 °C was required to reach the abovementioned 8.0 V, and the corresponding hot- and cold-end temperatures were 71 °C and 39 °C, respectively. The input voltage *U*_in_ dropped to 5.9 V immediately because water pumps consumed electric power.

A possible solution to self-startupof the SPTEG without a battery is to install enough aluminum plates adjacent to water-cooled heat sinks. A large heat capacity augments the temperature difference after ignition [25], and once the open-circuit voltage generated by the SPTEG reaches 8.0 V, DDC-1 can be turned on. Four water pumps consumed 7.18 W when the working voltage was 6.0 V, which is comparable with that of air-cooling fans in [4], and is larger than that in [26].

As shown in Figure 4a, the hot-end temperature increased continuously, from 72 °C to 229 °C within 1500 s. However, the cold-end temperature remained relatively low, i.e., less than 59 °C. Therefore, the input voltage to the DDCs increased gradually from 5.9 V to 40.9 V, which resulted from the augmented temperature difference of 170 °C. An amount of 8.18 V per TE module was reached, and this value was underestimated since 7.18 W of electric power was consumed by four water pumps.

Figure 4b presents the time history of a self-startup process. As shown in Figure 1a, eight solid aluminum plates with dimensions of 200 mm × 80 mm × 8 mm were installed adjacent to water-cooled heat sinks. A total of 882 s were needed for the open circuit voltage to reach 8.0 V, and DDC-1 was turned on thereafter, powering up four water pumps. In this run of the SPTEG, a temperature difference of 31 °C was needed for self-startup. Once water pumps were initialized, the input voltage to the DDCs increased sharply within seconds, i.e., from 6.1 V to 13.5 V within 5 s, and in the meantime, the cold-end temperature dropped gradually. Due to the growth of the combustion, the input voltage, temperature difference, and hot- and cold-end temperatures increased thereafter. The maximum open-circuit voltage reached 42.5 V and the corresponding temperature difference was 173 °C. Comparing Figure 4a,b, the temperature results and the open-circuit voltage are consistent with each other, indicating good repeatability in different runs.

The temperature distributions of the heat collector are shown in Figure 4c, corresponding to the steady state of the run of Figure 4b. The hot-end temperatures cannot be maintained constantly, and decrease slightly. As expected from the design, temperature distributions of all four “ears” are comparable with each other (shown in Figure 4c), which is the essential foundation to connect all eight TE groups to DDCs in parallel. In other words, this type of heat collector and wiring method is able to install a large number of TE modules compactly and to save the number of DDCs. Similar designs, e.g., six “ears” in a hexagonal heat collector or eight “ears” in an octagonal heat collector, can be expected to work efficiently.

### 3.2. Power Load Feature and Cost Analysis

**Figure 5:**
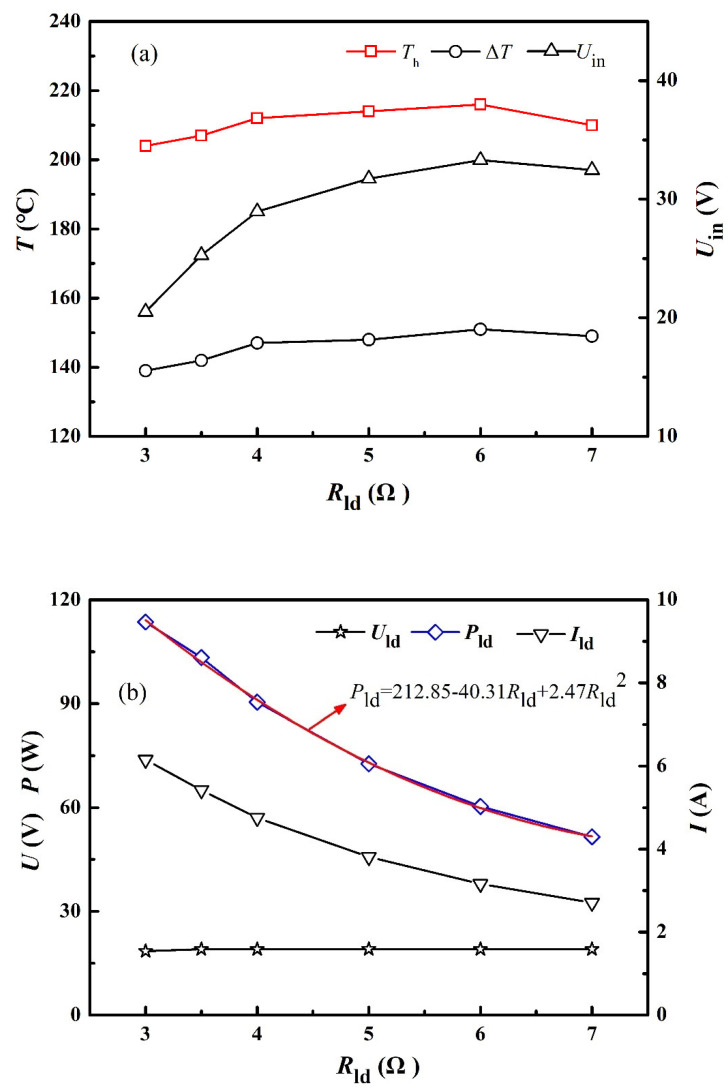

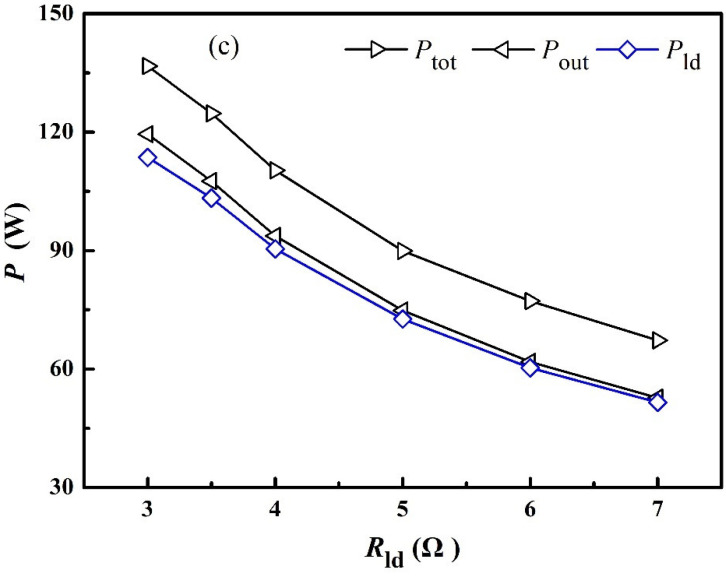
Power load feature when the output voltage is maintained at 19 V. (**a**) Input voltage and temperature. (**b**) Output voltage, load current, and load power. (**c**) Total power, output power, and load power.

Figure 5 depicts various parameters (*T*_h_, Δ*T*, *U*_in_, *U*_ld_, *I*_ld_, *P*_ld_, *P*_out_, and *P*_tot_) under different load resistances, and these results help to find out the optimized running conditions for maximum output power. *P*_tot_ represents the total generated electric power by TE modules, whereas *P*_out_ is the net electric power (the total electric power minus the power consumption by water pumps). The temperature difference varies between 140 °C and 153 °C during experiments. The hot-end temperature decreases slightly as the load resistance drops, and this phenomenon is the result of the Peltier effect, i.e., pumping more and more heat to the cold-end side because a lower load resistance results in a larger current. As shown in Figure 5, the total power, output power, and load power could be augmented by adjusting the load resistance, but too small load resistance will cause unstable output voltage. The obtained maximum total power, output power, and load power are 137 W, 119 W, and 113 W, respectively. The DDCs and water pumps consume a total of 18 W, corresponding to the difference between the abovementioned 137 W and 119 W. As shown in Figure 5c, line loss has to be taken into consideration. The line loss is 5.9 W when the maximum power output was extracted, and this is caused by the large load current (6.16 A).

An empirical equation, correlating the load power and load resistance on the basis of a correlation coefficient larger than 95%, shown in Figure 5b, depicts the exponential decreasing trend of the load power, as follows:

(1) $$P_{\mathrm{ld}}=25.828+226.542e^{-\left(R_{ld}/3.198\right)}$$

when 3 Ω ≤ *R*_ld_ ≤ 7 Ω.

Furthermore, it is possible to adjust the output voltage to other values. However, the SPTEG performance is affected, and 19.0 V is an optimized setting for the present SPTEG after several tests (12 V, 15 V, and 24 V). In the case of setting the output voltage to 12.0 V, only 124 W could be reached, and the corresponding output power and load power are 103 W and 93 W, respectively. The load power can be correlated by the following equation when the output voltage is 12.0 V on the basis of a correlation coefficient larger than 99%:

(2) $$P_{\mathrm{ld}}=20.108+184.612e^{-\left(R_{ld}/1.607\right)}$$

when 1.5 Ω ≤ *R*_ld_ ≤ 6 Ω.

Concerning the cost of the present SPTEG, the present type of TE module can be as low as CNY 4.29 per unit in the open market (Alibaba.com accessed on 3 December 2021). As a result, $172 is needed for the component of TE modules, whereas approximate CNY 180 is needed for copper plates, heat sinks, water pumps, DDCs, voltage meters, and other accessories. Therefore, the material cost of the present SPTEG is approximately CNY 352, which corresponds to a material cost of CNY 3.11/W based on the load electric power of 113 W. Extensive production could cut down the material cost.

### 3.3. Power Generation Discussions

**Figure 6:**
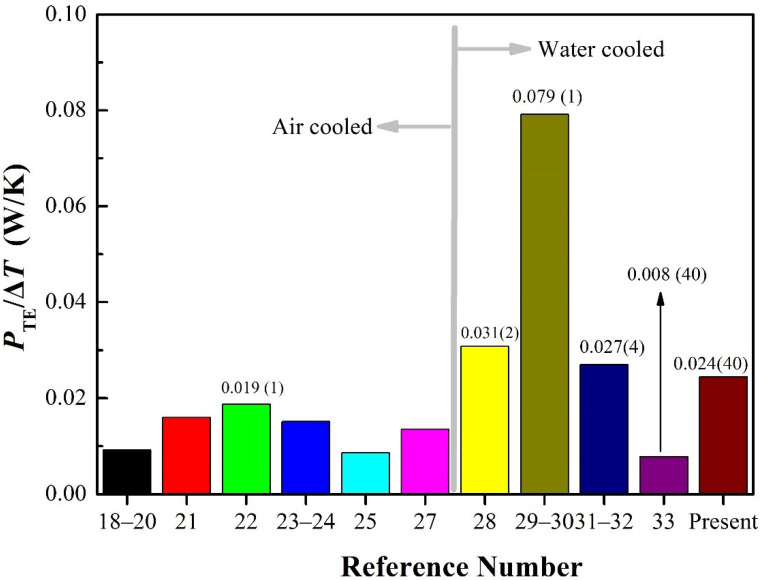
Comparison of *P*_TE_/Δ*T* with various SPTEGs. The number of TE modules is given inside the parentheses.

Figure 6 shows the *P*_TE_/Δ*T* of various SPTEGs, where *P*_TE_ represents the generated electric power from an individual TE module, and *P*_TE_/Δ*T* reveals the scientific nature of the whole SPTEG system. As shown in Figure 6, previous studies confirmed each other except [29,30], which had a much better performance compared with other SPTEGs, and this should be further confirmed.

The *P*_TE_/Δ*T* of air-cooled SPTEGs is lower than those of water-cooled SPTEGs when the number of TE modules is comparable. For example, the *P*_TE_/Δ*T* in [31,32] (water-cooled SPTEG) with four TE modules equals 0.027 W/K, which is considerably larger than that (0.019 W/K) in [22] (air-cooled SPTEG) where only one TE module was employed.

It is interesting to explore the change trend of *P*_TE_/Δ*T* when the number of TE modules increases. The SPTEG in [28] adopted two TE modules, generating electricity of 0.031 W/K per TE module. The SPTEG in [31,32] adopted four TE modules, generating electricity of 0.027 W/K per TE module, which is decreased by 12.9% compared with that of [28]. In the case of forty TE modules incorporated in [33], the *P*_TE_/Δ*T* decreased sharply to 0.008 W/K, indicating that the SPTEG in [33] has the potential to be further optimized. In the present work, the number of TE modules is also forty, but the *P*_TE_/Δ*T* has increased to 0.024 W/K, which is 200% higher compared with [33] and is only 11% lower compared with [31,32] even though the number of TE modules is increased to ten times of that in [31,32]. This is achieved by the proper design of the novel heat collector. Nevertheless, the *P*_TE_/Δ*T* is not as high as those in previous SPTEGs with several TE modules [28,31,32]. The reason for the abovementioned performance downgrade is the internal power loss [35], which is caused by the uneven temperature distribution of the heat spreader when multiple TE modules were involved [35]. Hence, it is of vital importance to develop a proper heat collector to increase *P*_TE_/Δ*T*in addition to installing sufficient TE modules in future studies.

### 3.4. Conversion Efficiency

The heat dissipation rate by the cooling water has to be determined to obtain the TE efficiency, as follows:

(3) $$Q_{\mathrm{out}}=c_{p}m_{}(T_{\mathrm{out}}-T_{in}),$$

where *m* is the mass flow rate of cooling water, which is measured with the weighing method. Thus, the TE efficiency can be calculated as follows:

(4) $$\xi =\frac{P_{\mathrm{tot}}}{P_{\mathrm{tot}}+Q_{\mathrm{out}}}$$

The total electric power is 137 W. The inlet water temperature and the outlet water temperature are 29 °C and 37 °C, respectively. The mass flow rate of cooling water is 0.1265 kg/s. As a result, the heat dissipation rate into the cooling water is 4250 W. Hence, the TE efficiency reaches 3.12%, and the related measurements are summarized in Table 4.

It should be noted that TE efficiency is not the overall efficiency of the whole SPTEG. To the best of our knowledge, few previous SPTEG works reported the overall efficiency of the whole SPTEG, and only TE efficiency can be measured and discussed in the present work. The relationship between TE efficiency and overall efficiency can be found in our recent work [36]. Table 5 presents previously reported TE efficiencies. Improving TE material is vital to accelerate the application of SPTEG, e.g., PbTe [7] and half-Heusler [37] are some promising TE materials. On the other hand, increasing the working temperature is another way to augment the SPTEG performance, yet special attention should be played because the aging problem is another important issue for an SPTEG. Multistage TEG is another possible method to increase TE efficiency of the entire SPTEG [38].

### 3.5. Influence of Water Flow Rate on SPTEG Performance

The water flow rate, *m*, can be adjusted by conditioning the input voltage of water pumps. Increasing *m* improves heat dissipation rate of the SPTEG, but consumes more electric power. Therefore, the enhanced power generation and increased internal power consumption are interactive. Table 6 presents the experimental cases to optimize the water flow rate. Two power ratios are defined as follows:

(5) $$P{*}_{\mathrm{tot}}=\frac{P_{\mathrm{tot}}}{P_{\mathrm{tot}}at5.0V},$$

(6) $$P{*}_{\mathrm{out}}=\frac{P_{\mathrm{out}}}{P_{\mathrm{out}}at5.0V},$$

where P*_tot_ is the total electric power ratio, while P*_out_ is the electric power output ratio. During experiments in this section, DDC-2 shown in Figure 2 was not used, and the input lines were directly connected to the electronic load instrument.

**Figure 7:**
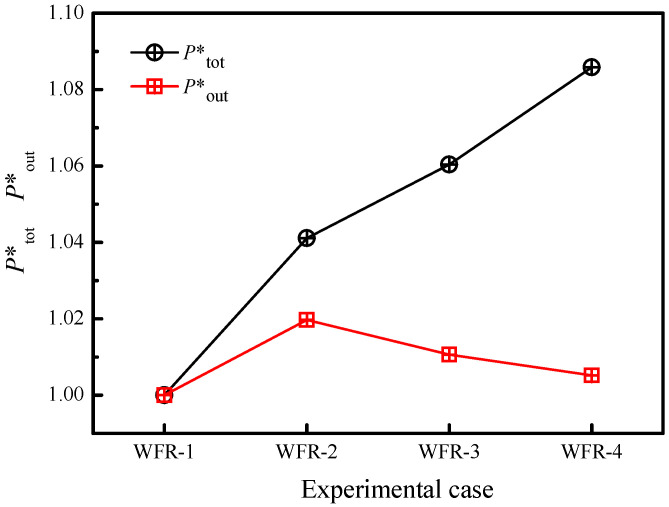
Influence of the water mass flow rate on SPTEG performance.

Figure 7 presents the influence of the water mass flow rate on the total electric power ratio and the electric power output ratio. The cooling water mass flow rate has a minor impact on the electric power output ratio (less than 2%) although the total electric power was augmented slightly (less than 9%). This is caused by the increased internal power consumption by water pumps, as is shown in Table 6. The maximum electric power output ratio is 1.019 in the case of WFR-2, indicating the enhancement is negligible. It should be noted that different water pumps may exhibit different performances. However, the present study reveals that efforts to optimize the water flow rate aiming to increase the electric power output may be insignificant. Other methods, such as flow-impeding inserts [39], flow pulsation [40], and flow impinging [41], are some examples to enhance the heat sink performance. Besides, heat pipes could be adopted to enhance the heat distribution [42].

## 4. Conclusions

A water-cooled, stove-powered high capacity thermoelectric generator (SPTEG) incorporated with a novel heat collector was designed. The SPTEG startup, power generation performance, and conversion efficiency were explored in detail. The influence of water flow rate on SPTEG performance was studied. A one-dimensional theoretical model was employed to analyze the SPTEG performance. Several conclusions can be drawn as follows:

(1) For the present SPTEG, a maximum electric power of 137 W is generated, and 119 W could be outputted to the external load at the stabilized voltage of 19 V. The TE efficiency of the present SPTEG is 3.12% at the temperature difference of 140 °C.
(2) It is of vital importance to develop a proper heat collector to augment the capacity of an SPTEG in addition to installing sufficient TE modules. Forty TE modules are able to be installed in the present SPTEG owing to the novel heat collector. The developed SPTEG is able to generate comparable electricity from each TE module even though the number of TE modules is ten times that of previous SPTEGs.
(3) Increasing the mass flow rate of the cooling water augments the total generated electric power, but minor augments can be found related to the electric power output to the external load.

## Figures and Tables

**Table 1:** Detailed comparisons of previous SPTEGs (*P*_out_ is the net power excluding the power consumption by water pumps and Δ*T* is the temperature difference between the hot-end and the cold-end of the TE module).

Authors	Year	Cooling Method	*P*_out_/*P*_tot_ (W)	TE Num.	Δ*T* (°C)
Mal et al. [4]	2016	Air-cooled	4.0/6.0	2	−
Killander and Bass [5]	1996	Air-cooled	−/10	2	−
Nuwayhid et al. [18,19,20]	2003–2005	Air-cooled	4.2/4.2	3	152
Lertsatitthanakorn [21]	2007	Air-cooled	2.4/2.4	1	150
Raman [22]	2014	Air-cooled	3.7/4.5	1	240
Najjar and Kseibi [23,24]	2016–2017	Air-cooled	7.8/7.8	12	43
O’Shaughnessy et al. [25]	2015	Air-cooled	3.0/5.9	1	350
BioLiteBaseCamp [26]	2018	Air-cooled	−/5	2	−
Li et al. [27]	2018	Air-cooled	4.7/12.9	8	119
Rinalde et al. [28]	2010	Water-cooled	−/12.3	2	200
Champier et al. [29,30]	2010–2011	Water-cooled	7.6/9.5	1	120
Montecucco et al. [31,32]	2015–2017	Water-cooled	19/27	4	250
Sornek et al. [22]	2019	Water-cooled	−/75.2	40	240
Present		Water-cooled	119/137	40	140

**Table 2:** TE properties of SAGREON 12708.

Parameter	Unit	Value
α_P_	V/K	223.2 × 10^−6^
α_N_	V/K	−187.7 × 10^−6^
ρ_P_	Ωm	1.83 × 10^−5^
ρ_N_	Ωm	1.58 × 10^−5^

**Table 3:** Measuring errors and deduced parameter errors.

Parameter	Error (%)	Parameter	Error (%)
*U*	±0.1	*I*	±0.1
*P*	±0.2	*T*	±0.5
*m*	±2.0	*Q*_out_	±3.0
*ξ*_DDC_	±0.4	*ξ*	±3.4

**Table 4:** Measurements for TE efficiency.

Parameter	Value	Parameter	Value
*P*_tot_(W)	137	*T*_out_ (°C)	37
*T*_h_(°C)	204	m (kg/s)	0.1265
*T*_c_(°C)	64	*Q*_out_(W)	4250
*T*_in_ (°C)	29	*ξ* (%)	3.12

**Table 5:** TE efficiency of various SPTEGs.

Δ*T* (K)	TE Material	*ξ*	Cooling Method	Reference
150	Bi_2_Te_3_	3.2%	Air-cooled	[21]
200	Bi_2_Te_3_	2%	Water-cooled	[29,30]
150–200	Bi_2_Te_3_	4–5%	Water-cooled	[31,32]
140	Bi_2_Te_3_	3.12%	Water-cooled	Present

**Table 6:** Experimental cases for optimization of the water flow rate.

No.	*U*_p_ (V)	*P*_p_ (W)	*m* (Kg/s)
WFR-1	5.0	5.10	0.085
WFR-2	6.0	7.18	0.127
WFR-3	7.0	9.75	0.145
WFR-4	8.0	12.58	0.159

## Data Availability

The data presented in this study are available on request from the corresponding author.

## Nomenclature

*I*	Current (A)
*I*_ld_	Load current (A)
*m*	Mass flow rate of cooling water (kg/s)
*P*	Power (W)
*P*_ld_	Load electric power (W)
*P*_out_	Electric power output (W)
*P*_tot_	Total electric power (W)
*P*_TE_	Electric power generated per TE module (W)
*P**_out_	Electric power output ratio (dimensionless)
*P**_tot_	Total electric power ratio (dimensionless)
*R*_ld_	Load resistance (Ω)
*T*	Temperature (°C)
*T*_c_	Cold-end temperature (°C)
*T*_h_	Hot-end temperature (°C)
*T*_in_	Inlet water temperature (°C)
*T*_out_	Outlet water temperature (°C)
Δ*T*	Temperature difference (°C), ΔT = T_h_−T_c_
*U*	Voltage (V)
*U*_in_	Input voltage to the DDC (V)
*U*_ld_	Output voltage (V)
*ξ*	TE efficiency (%)
*ξ*_DDC_	Transform efficiency of the DDC (%)
Abbreviation	
DAQ	Data-acquisition
DDC	DC–DC converter
EET	Electric energy tester
MPPT	Maximum power point tracking
SPTEG	Stove-powered TE generator
TE	Thermoelectric
TEG	TE generator
